# The positive association between evening or night work schedules and coronary heart disease or angina among U.S. adults: A cross-sectional study

**DOI:** 10.1016/j.ajpc.2025.101270

**Published:** 2025-08-23

**Authors:** Jingyi Ding, Yuzhi Jia, Ran Ji, Hui Zhang, Ziyi Wang, Xinbin Song, Jing Gao, Qingyong He

**Affiliations:** a Department of Cardiology, Guang’anmen Hospital, China Academy of Chinese Medical Sciences, Beijing, PR China; b Department of Intensive Care Unit, Guang’anmen Hospital, China Academy of Chinese Medical Sciences, Beijing, PR China; c Hubei University of Chinese Medicine, Hubei, Wuhan, PR China; d Department of Intensive Care Unit, Zhumadian Hospital of Traditional Chinese Medicine, Zhumadian, Henan, PR China

**Keywords:** Coronary heart disease, Angina, Work schedules, Cross-sectional study, NHANES

## Abstract

**Background:**

Currently, there is a scarcity of research exploring the connection between work schedules and coronary heart disease (CHD) or angina. Previous studies on the associations between work schedules and CHD or angina have been primarily limited to specific occupations, particular genders, small sample sizes, or narrow regional focus. This study aims to evaluate the potential association between work schedules and CHD or angina among adults in the United States.

**Methods:**

In this cross-sectional study, we selected 13,147 adults aged ≥ 20 years from the National Health and Nutrition Examination Survey 2005–2010 and 2017–2020 cycles. We computed adjusted odds ratio (OR) and 95 % confidence interval (CI) utilizing multivariate logistic regression models. Meticulous subgroup analyses were conducted to ensure the reliability and consistency of our findings. Propensity score assessments were implemented to enhance the comparability between daytime workers and evening or night workers, thereby facilitating a more accurate estimation.

**Results:**

Among the participants, 216 were diagnosed with CHD, while 125 were diagnosed with angina. Evening or night workers exhibited a prevalence of CHD that was 1.87 times higher than that of daytime workers (OR: 1.87, 95 % CI: 1.19–2.95, *P* = 0.007). Furthermore, evening or night workers exhibited a prevalence of angina that was 1.81 times higher than that of daytime workers (OR: 1.81, 95 % CI: 1.05–3.12, *P* = 0.033). Our findings demonstrated robustness and reliability through subgroup analyses and propensity score assessments.

**Conclusions:**

In conclusion, evening or night work schedules were associated with increased CHD and angina risk. Further research should explore biological mechanisms for prevention in this population.

## 1 Introduction

Coronary Heart Disease (CHD) remains the leading cause of global mortality [1], accounting for 33 % of deaths worldwide [2,3]. Its common symptom, angina, reflects underlying CHD and contributes significantly to healthcare burdens, particularly in the United States (U.S.).

Societal and economic demands have driven an increased need for 24-hour work schedules in various industries. Evening or night work disrupts circadian rhythms, leading to irregular sleep-wake cycles that heighten work-related stress [4]. Consequently, evening or night work is acknowledged as a contributing factor to cardiovascular diseases (CVDs) [5]. Evidence indicates that evening or night workers have higher CVD incidence than daytime workers, including increased prevalence and severity of hypertension, left ventricular hypertrophy, and myocardial infarction [6], along with increased inflammation, dyslipidaemia, and impaired cardiac excitability [7]. Among hypertensive patients, these workers also exhibit a 16 % higher risk of cardiometabolic multimorbidity [8]. A systematic meta-analysis further demonstrated that evening or night workers have the highest coronary event risk compared to other shift types [9].

Previous population-based studies have identified associations between evening or night work and CHD, angina, or ischemic heart disease in specific occupational groups [[10], [11], [12]]. However, these studies were either limited to particular populations (e.g., nurses) or failed to distinguish between CHD and angina diagnoses. Additionally, several cross-sectional studies were limited to narrow sample sizes [13,14]. To address these limitations, we utilized a cross-sectional study based on data from the National Health and Nutrition Examination Survey (NHANES) to investigate the relationship between work schedules and the prevalence of CHD or angina among U.S. workers.

## 2 Methods

### 2.1 Data sources and study participants

NHANES is a nationally representative survey administered by the National Center for Health Statistics of the Centers for Disease Control and Prevention. The study employed a sophisticated, stratified, multistage probability cluster methodology to collect data from the noninstitutionalized U.S. population [15]. All participants furnished written informed consent before participating in NHANES. This study followed the Strengthening the Reporting of Observational Studies in Epidemiology reporting guidelines.

**Fig. 1:**
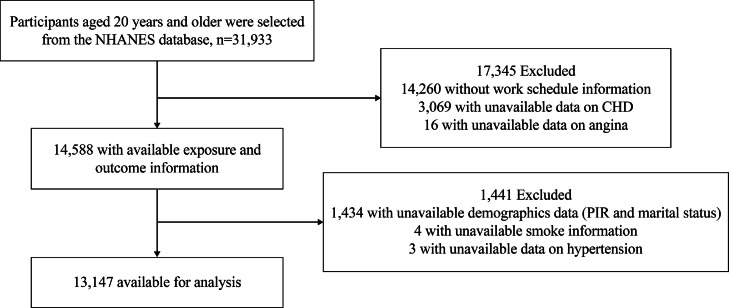
The flow chart of the screening and enrollment of study participants. NHANES, National Health and Nutrition Examination Survey; CHD, coronary heart disease; RIR, poverty income ratio.

In this cross-sectional study, information on work schedules was available only in the NHANES 2005–2010 and 2017–2020 cycles for adults aged ≥ 20 years, forming the basis for our analysis. The following exclusion criteria were applied: age < 20 years; unavailable data on work schedules, CHD, and angina; and unavailable demographic information (including age, sex, race/ethnicity, marital status, and poverty income ratio [PIR]), as well as data on physical activity (PA), smoking status, and hypertension. The data analysis was conducted on 13,147 participants (Fig. 1).

### 2.2 Work schedules

Information about work schedules was gathered in participants' homes by skilled interviewers utilizing the computer-assisted personal interview system, which is programmed with built-in consistency checks to reduce data entry errors and assisted interviewers in defining key terms used in the questionnaire. Based on the occupation questionnaire regarding work schedules, participants were categorized into three groups: (a) Daytime worker (including persons who answered “A regular daytime schedule”, “Traditional 9 AM to 5 PM day”, or “Early mornings”); (b) Evening or night worker (including persons who answered “A regular evening shift”, “A regular night shift”, or “Evening or nights”); and (c) Shift worker (including persons who answered “A rotating shift”, “Another schedule”, or “Variable [early mornings, days, and nights]”).

### 2.3 Diagnosis of CHD or angina

According to previous NHANES research, CHD is primarily diagnosed based on a history of CHD [16,17], and angina is mainly diagnosed based on a history of angina. If participants responded affirmatively to the query, "Have you ever been informed by a doctor or other health professional that you had CHD?" or "Have you ever been informed by a doctor or other health professional that you had angina?" they were considered to be diagnosed with CHD or angina.

### 2.4 Covariate assessment

Details on age, sex, race/ethnicity, marital status, PIR, PA, smoking status, and hypertension history were obtained from questionnaires and assessed according to the literature [[18], [19], [20]]. Covariates were categorized as follows: race/ethnicity (non-Hispanic White, non-Hispanic Black, Mexican American, other Hispanic, and other race), marital status (married, never married, living with a partner, and other [including widowed, divorced, or separated]), PIR (low income [PIR ≤ 1.30], medium income [PIR = 1.31–3.50], and high income [PIR > 3.50]) [21], and smoking status (never [less than 100 cigarettes], former [more than 100 cigarettes but quit], and current [more than 100 cigarettes and currently smoke]). Participants were considered diagnosed with hypertension if they responded "yes" to the question, "Have you ever been told by a doctor or other health professional that you have hypertension, also called high blood pressure?" or "Are you now taking prescribed medicine for high blood pressure?".

### 2.5 Statistical analysis

Mean and standard deviation were utilized for continuous variables with a normal distribution; otherwise, median values and interquartile ranges were utilized to characterize the central tendency and spread of non-normally distributed continuous variables. Kruskal-Wallis tests and *χ^2^* tests were employed to compare continuous and categorical data, respectively. To explore the association between work schedules and CHD or angina, multivariate logistic regression models were employed, determining the odds ratio (OR) and 95 % confidence interval (CI). Age adjustment was made in Model 1, while Model 2 incorporated adjustments for factors from Model 1 and sex, race/ethnicity, and marital status. Model 3 went a step further, encompassing adjustments for factors from Model 2 and PIR, PA, smoking status, and hypertension.

Furthermore, interaction and subgroup analyses were conducted to assess the stability of the relationship between work schedules and CHD or angina across different populations. These analyses were performed by age (< 40 years, 40–59 years, and ≥ 60 years) [22], sex, PIR (low income [PIR ≤ 1.30], medium income [PIR = 1.31–3.50], and high income [PIR > 3.50]) [21], and smoking status (non-smokers versus smokers). Logistic regression models and likelihood ratio tests were used to assess heterogeneity and interactions between subgroups.

Propensity score matching (PSM) [23] was conducted for evening or night workers and daytime workers with a multivariable logistic regression model. A 1:1 nearest neighbor matching algorithm was employed with a caliper width of 0.2. Standardized mean differences were used to evaluate the balance after PSM, using age, sex, race/ethnicity, marital status, PIR, PA, smoking status, and hypertension as model covariates for propensity score assessments. Propensity scores were used as weights. To ensure robustness, the inverse probability of treatment weighting (IPTW) [24], the pairwise algorithm [25], and the overlap weight [26] models were used to address potential confounding factors. All statistical analyses were conducted using Free Statistics software (version 1.9.2) and the statistical software programs R (version 4.1.2; https://www.r-project.org/).

## 3 Results

### 3.1 Participant selection and general characteristics

The current study comprised a total of 31,933 participants from NHANES, with 13,147 participants included (Fig. 1). Exclusions were made for 17,345 participants due to missing or implausible data for work schedules, CHD, or angina, resulting in 14,588 participants available for analysis. An additional 1441 participants lacking covariate data were also excluded.

The baseline characteristics of the study participants are outlined in Table 1. The median age of the participants included was 42.9 (28.8–57) years, and 53.1 % were male. A total of 1333 participants worked in the evening or at night. Among all participants, 216 were diagnosed with CHD, while 125 were diagnosed with angina.

**Table 1:** Baseline characteristics of study participants according to work schedules.

Variables	Total (*n* = 13,147)	Daytime worker (*n* = 8826)	Evening or night worker (*n* = 1333)	Shift worker (*n* = 2988)	*P* value
**Age, Mean ± SD**	42.9 ± 14.1	43.4 ± 13.7	39.8 ± 14.3	42.9 ± 15.1	< 0.001
**Age, n ( %)**					< 0.001
<60	11,171 (85.0)	7509 (85.1)	1177 (88.3)	2485 (83.2)	
≥60	1976 (15.0)	1317 (14.9)	156 (11.7)	503 (16.8)	
**Sex, n ( %)**					0.075
Male	6979 (53.1)	4628 (52.4)	713 (53.5)	1638 (54.8)	
Female	6168 (46.9)	4198 (47.6)	620 (46.5)	1350 (45.2)	
**Race/Ethnicity, n ( %)**					< 0.001
Non-Hispanic White	5530 (42.1)	3820 (43.3)	437 (32.8)	1273 (42.6)	
Non-Hispanic Black	2918 (22.2)	1782 (20.2)	393 (29.5)	743 (24.9)	
Mexican American	2230 (17.0)	1641 (18.6)	210 (15.8)	379 (12.7)	
Other Hispanic	1194 (9.1)	813 (9.2)	142 (10.7)	239 (8)	
Other race	1275 (9.7)	770 (8.7)	151 (11.3)	354 (11.8)	
**Marital Status, n ( %)**					< 0.001
Married	7524 (57.2)	5264 (59.6)	613 (46)	1647 (55.1)	
Never married	2686 (20.4)	1582 (17.9)	394 (29.6)	710 (23.8)	
Living with partner	793 (6.0)	569 (6.4)	100 (7.5)	124 (4.1)	
Other status	2144 (16.3)	1411 (16)	226 (17)	507 (17)	
**PIR, Mean ± SD**	2.9 ± 1.6	3.0 ± 1.6	2.4 ± 1.5	2.8 ± 1.7	< 0.001
**PIR, n ( %)**					< 0.001
≤1.30	2881 (21.9)	1752 (19.9)	381 (28.6)	748 (25)	
1.31–3.50	5030 (38.3)	3305 (37.4)	617 (46.3)	1108 (37.1)	
>3.50	5236 (39.8)	3769 (42.7)	335 (25.1)	1132 (37.9)	
**PA, Mean ± SD**	1063.4 ± 1774.6	952.8 ± 1675.6	1302.5 ± 1921.8	1283.4 ± 1951.3	< 0.001
**Smoke, n ( %)**					< 0.001
Never	7714 (58.7)	5230 (59.3)	735 (55.1)	1749 (58.5)	
Former	2723 (20.7)	1876 (21.3)	218 (16.4)	629 (21.1)	
Now	2710 (20.6)	1720 (19.5)	380 (28.5)	610 (20.4)	
**CHD, n ( %)**					0.397
No	12,931 (98.4)	8690 (98.5)	1307 (98)	2934 (98.2)	
Yes	216 (1.6)	136 (1.5)	26 (2)	54 (1.8)	
**Angina, n ( %)**					0.259
No	13,022 (99.0)	8750 (99.1)	1316 (98.7)	2956 (98.9)	
Yes	125 (1.0)	76 (0.9)	17 (1.3)	32 (1.1)	
**Hypertension, n ( %)**					0.128
No	9403 (71.5)	6275 (71.1)	983 (73.7)	2145 (71.8)	
Yes	3744 (28.5)	2551 (28.9)	350 (26.3)	843 (28.2)	

CHD, coronary heart disease; PIR, poverty income ratio; PA, physical activity; SD, standard deviation.

### 3.2 Association between work schedules and CHD or angina prevalence

In multivariable logistic regression analysis adjusted for age, sex, race/ethnicity, marital status, PIR, PA, smoking status, and hypertension in Model 3, evening or night work schedules were significantly positively associated with CHD or angina compared to daytime work schedules. The prevalence of CHD was 1.87 times higher in evening or night workers than in daytime workers (OR: 1.87, 95 % CI: 1.19–2.95, *P* = 0.007) (Table 2, Model 3). Moreover, the prevalence of angina was 1.81 times higher in evening or night workers than in daytime workers (OR: 1.81, 95 % CI: 1.05–3.12, *P* = 0.033) (Table 2, Model 3). Nevertheless, there was no significant difference in the prevalence of CHD or angina between daytime workers and shift workers.

**Table 2:** Association between work schedules and CHD or Angina.

	Cases/participants	Model 1^a^		Model 2 b		Model 3 c	
		OR (95 % CI)	*P* value	OR (95 % CI)	*P* value	OR (95 % CI)	*P* value
**CHD**							
Daytime worker	8826/13,147	1 [Reference]		1 [Reference]		1 [Reference]	
Evening or night worker	1333/13,147	1.66 (1.07–2.56)	0.024	1.84 (1.18–2.87)	0.007	1.87 (1.19–2.95)	0.007
Shift worker	2988/13,147	1.04 (0.75–1.44)	0.83	0.99 (0.71–1.39)	0.966	1.06 (0.76–1.49)	0.732
**Angina**							
Daytime worker	8826/13,147	1 [Reference]		1 [Reference]		1 [Reference]	
Evening or night worker	1333/13,147	1.78 (1.04–3.03)	0.034	1.88 (1.09–3.21)	0.022	1.81 (1.05–3.12)	0.033
Shift worker	2988/13,147	1.2 (0.79–1.82)	0.399	1.88 (1.09–3.21)	0.022	1.22 (0.8–1.86)	0.359

CHD, coronary heart disease; CI, confidence interval; OR, odds ratio.

a Adjusted for age.

b Adjusted for age, gender, race/ethnicity, and marital status.

c Adjusted for age, gender, race/ethnicity, marital status, poverty income ratio, physical activity, smoking status, and hypertension.

### 3.3 Subgroup analyses

**Fig. 2:**
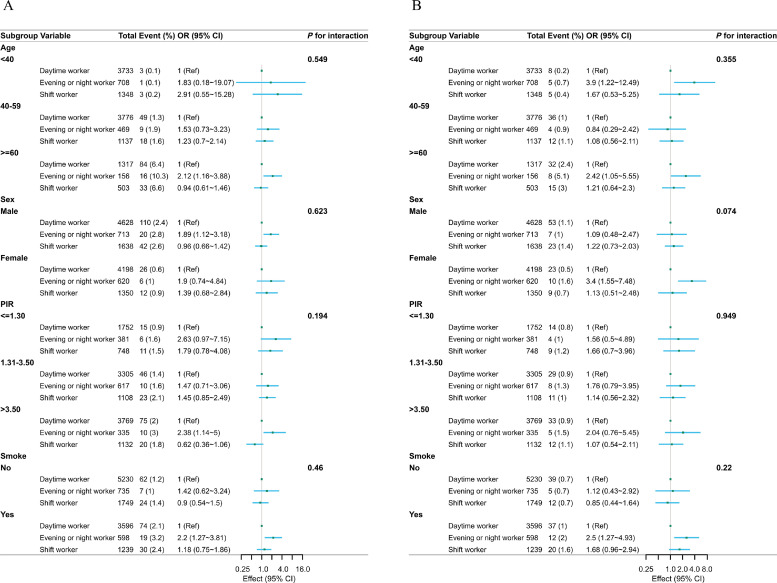
Subgroup analyses. (A) Subgroup analyses of the association between work schedules and coronary heart disease. (B) Subgroup analyses of the association between work schedules and angina. OR, odds ratio; Ref, reference group.

Fig. 2 illustrates the outcomes of the subgroup analyses. Subgroup analyses aimed to investigate the potential connection between work schedules and the prevalence of CHD (Fig. 2A) or angina (Fig. 2B) across various subgroups categorized by age, sex, PIR, and smoking status. However, no distinctions were observed among all subgroups regarding the influence of work schedules on CHD or angina prevalence.

### 3.4 Propensity score assessment

**Fig. 3:**
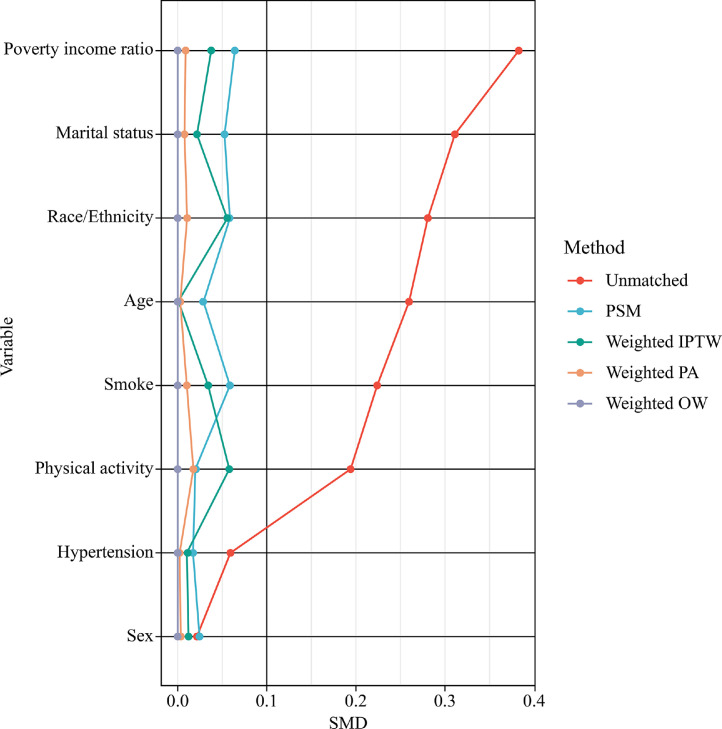
Standardized mean difference between evening or night workers and daytime workers. PSM, propensity score matched; IPTW, inverse probability of treatment weighting; PA, pairwise algorithmic; OW, overlap weight; SMD, standardized mean difference.

After PSM, the 1333 pairs (one from each group) were well-matched, displaying no significant differences between the two matched groups (Fig. 3). Various methods of propensity score assessments indicated that the prevalence of CHD and angina remained higher in evening or night workers when compared to daytime workers. The OR for CHD prevalence was 2.63 (95 % CI: 1.26–5.48, *P* = 0.01) after PSM. The OR for angina prevalence was 2.45 (95 % CI: 1.01–5.92, *P* = 0.047) after PSM. After IPTW, the ORs were 1.77 (95 % CI: 1.21–2.6, *P* = 0.003) for CHD prevalence and 2.08 (95 % CI: 1.29–3.34, *P* = 0.003) for angina (Table 3).

**Table 3:** The association between evening or night workers and CHD or angina compared to daytime workers after matching.

Analysis	CHD		Angina	
	OR (95 % CI)	*P* value	OR (95 % CI)	*P* value
Multivariable Adjusted	1.91 (1.21–3.01)	0.006	1.87 (1.08–3.24)	0.025
Propensity Score Adjusted	1.99 (1.29–3.08)	0.002	1.96 (1.14–3.37)	0.016
PSM	2.63 (1.26–5.48)	0.01	2.45 (1.01–5.92)	0.047
Weighted IPTW	1.77 (1.21–2.6)	0.003	2.08 (1.29–3.34)	0.003
Weighted PA	1.98 (1.02–3.85)	0.044	1.94 (0.86–4.38)	0.112
Weighted OW	1.95 (0.96–3.95)	0.065	1.93 (0.81–4.62)	0.139

CHD, coronary heart disease; CI, confidence interval; OR, odds ratio; PSM, propensity score matched; IPTW, inverse probability of treatment weighting; PA, pairwise algorithmic; OW, overlap weight.

## 4 Discussion

The outcomes of this cross-sectional study indicate a consistent positive association between evening or night work schedules and the prevalence of CHD or angina when compared to daytime work schedules. Nonetheless, no significant disparity in the prevalence of CHD or angina was observed between daytime work schedules and shift work schedules. Our findings remained robust in subgroup analyses and propensity score assessments.

Prior prospective cohort studies conducted by Céline et al [10] and Kivimäki et al [11], each independently identified elevated CHD and angina risk among female nurses engaged in prolonged night shifts. While informative, these studies were limited by gender and occupational homogeneity. Our study included a variety of gender and occupational groups, thereby yielding more universally applicable conclusions. Additionally, Kivimäki et al.'s study treated angina only as a CVD screening criterion and Céline et al.'s study considered it merely a CHD symptom; our study specifically examined its independent association with work schedules to address this gap. Previous cross-sectional investigation involving 140 adults revealed that evening or night work was linked to the increased atherogenic index of plasma score [13], a well-established predictor of atherosclerosis progression and subsequent CHD risk. Our large-scale analysis strengthens this observation.

The increased prevalence of CHD or angina among evening or night workers may be mediated by elevated oxidative stress and chronic inflammation. Night workers exhibit higher Interleukin (IL)-1β and IL-6 levels compared to daytime workers [27,28]. Rotational or permanent night shifts elevate oxidative stress markers, including DNA damage, lipid peroxidation, and reactive oxygen species, while reducing repair capacity and antioxidant defenses [29]. These effects are compounded by circadian disruption, as night work interferes with the 24-hour biological rhythms governing sleep-wake cycles, metabolism, and cardiovascular functions [30]. The resulting metabolic and hormonal dysregulation also promotes inflammation [31], oxidative stress [28], hypertension [32], and dyslipidemia [32,33], collectively increasing CHD risk [[34], [35], [36]].

The potential pathophysiological mechanisms of the impact of evening or night work on CHD and angina are also tied to occupational stress [37]. Night-shift security guards exhibited higher salivary cortisol during street patrols [38], and elevated levels of late-night salivary cortisol indicate a heightened risk of coronary calcification [39] and cardiovascular mortality [40]. It can be inferred that the mental stress induced by evening or night work is also a significant contributing factor to the increased incidence of CHD or angina.

Research also underscored the impact of sleep quality on the occurrence of CHD and angina among evening or night workers, which carries significant implications for their overall health and well-being [[41], [42], [43]]. Overnight work is linked to difficulty falling asleep, sleep disturbances, sleep fragmentation, early morning awakening, and shorter, unconsolidated daytime sleep, all of which can lead to chronic sleep deprivation [44,45]. Suboptimal sleep behaviors are also associated with an increased risk of CHD or angina through diverse underlying mechanisms, including oxidative stress, inflammation, metabolic irregularities, changes in neural autonomic control, coagulatory responses, and sympathetic disturbance [46,47].

After a sudden change in work schedules, re-establishing rhythm synchronization can be accomplished by taking a short daytime nap [48]. Nevertheless, when this change persists for five days or more, it leads to a misalignment between the internal and external facets of the rhythm [49]. Besides, the risk of CHD morbidity was 26 % greater among shift workers compared to daytime workers, and this association was non-linear, becoming apparent only after five years of exposure [9]. The delayed effect observed in shift work might be attributable to its intermittent nature, which could provide recovery periods that mitigate acute physiological disruptions. This potential buffering effect could also explain why shift work schedules in our study did not exhibit a significant difference in the association with CHD or angina compared to daytime work schedules, whereas evening or night work schedules did.

Nonetheless, our study had limitations. First, the cross-sectional design precluded causal inference between work schedules and CHD or angina risk. Second, as highlighted in our supplemental analysis (supplementary table 1), the exclusion of 18,786 participants due to missing data introduced selection bias: excluded individuals were older and had higher baseline prevalence of hypertension, CHD, and angina, suggesting our included participants underrepresented higher-risk populations, which may have led to conservative effect estimates. However, the consistency between our primary analysis and PSM results reinforces the validity of our findings. Third, our study used NHANES questionnaire data, which has inherent limitations: former smoker was defined without consideration of cessation duration, and hypertension assessment based on self-reported diagnosis could underestimate true prevalence due to undiagnosed cases. Fourth, self-reported work schedules and disease history may introduce recall bias and exposure misclassification. Prior evidence suggests patients may recall their night shifts more accurately, potentially overestimating the estimated risk ratios [50]. However, if the inaccuracies occur equally across all groups, the bias may cancel out [51]. In our study, the use of PSM also helped to mitigate this bias. Importantly, our study lacked granular data on work intensity, actual hours, and occupational specifics—including shift work composition—which may explain the non-significant differences between shift workers and daytime workers. Large-scale prospective cohort studies are needed to address these limitations.

## 5 Conclusion

In this study, we observed a higher OR of CHD and angina among U.S. adults working in the evening or at night compared to those working during the day. The research emphasizes the necessity for intervention strategies to potentially diminish the risk of CHD or angina in evening or night workers. Moving forward, our findings should be validated in a prospective multi-ethnic study, and the underlying biological mechanisms contributing to the positive association between evening or night work schedules and CHD or angina should be further explored.

## Central illustration



## Declaration of generative AI in scientific writing

During the preparation of this work the authors used ChatGPT 3.5 in order to improve language and readability. After using this tool, the authors reviewed and edited the content as needed and take full responsibility for the content of the publication article.

## CRediT authorship contribution statement

**Jingyi Ding:** Writing – review & editing, Writing – original draft, Validation, Supervision, Methodology, Investigation, Formal analysis, Data curation, Conceptualization. **Yuzhi Jia:** Writing – review & editing, Writing – original draft, Visualization, Methodology, Conceptualization. **Ran Ji:** Writing – review & editing, Writing – original draft, Visualization, Methodology, Conceptualization. **Hui Zhang:** Writing – review & editing, Validation, Supervision, Methodology, Investigation. **Ziyi Wang:** Writing – review & editing, Validation, Supervision, Methodology, Investigation. **Xinbin Song:** Writing – review & editing, Validation, Investigation, Data curation. **Jing Gao:** Writing – review & editing, Validation, Investigation, Data curation. **Qingyong He:** Writing – review & editing, Funding acquisition, Conceptualization.

## Declaration of competing interest

The authors declare that they have no conflict of interests.

## Availability of data and materials

The data that support the findings of this study are available from the National Health and Nutrition Examination Survey (https://www.cdc.gov/nchs/nhanes/about_nhanes.htm)

## Appendix Supplementary materials

Image, application 1
