# Supplementary material for: The positive association between evening or night work schedules and coronary heart disease or angina among U.S. adults: A cross-sectional study
Source: Am J Prev Cardiol. 2025 Aug 23;24:101270. doi: 10.1016/j.ajpc.2025.101270 (PMC12510065; doi:10.1016/j.ajpc.2025.101270)
Supplement: Supplementary file 1 [file mmc1.docx]

Supplementary Table. Baseline characteristics of included and excluded participants.

| **Variables** | **Total (n = 31,933)** | **The included group**  **(n = 13,147)** | **The excluded group**  **(n = 18,786)** | ***P* value** |
| --- | --- | --- | --- | --- |
| **Work Schedules** |  |  |  | < 0.001 |
| Daytime worker | 11,388 (35.7) | 8826 (67.1) | 2562 (13.6) |  |
| Evening or night worker | 1857 ( 5.8) | 1333 (10.1) | 524 (2.8) |  |
| Shift worker | 4428 (13.9) | 2988 (22.7) | 1440 (7.7) |  |
| NA | 14,260 (44.7) | 0 (0) | 14,260 (75.9) |  |
| **Age, Mean ± SD** | 50.2 ± 18.1 | 42.9 ± 14.1 | 57.4 ± 18.7 | < 0.001 |
| **Sex, n ( %)** | | | | < 0.001 |
| Male | 12,782 (48.5) | 6979 (53.1) | 5803 (43.9) |  |
| Female | 13,582 (51.5) | 6168 (46.9) | 7414 (56.1) |  |
| **Race/Ethnicity, n ( %)** | | | | < 0.001 |
| Non-Hispanic White | 11,449 (43.4) | 5530 (42.1) | 5919 (44.8) |  |
| Non-Hispanic Black | 5931 (22.5) | 2918 (22.2) | 3013 (22.8) |  |
| Mexican American | 4233 (16.1) | 2230 (17) | 2003 (15.2) |  |
| Other Hispanic | 2392 ( 9.1) | 1194 (9.1) | 1198 (9.1) |  |
| Other Race | 2359 ( 8.9) | 1275 (9.7) | 1084 (8.2) |  |
| **Marital Status, n ( %)** | | | | < 0.001 |
| Married | 14,268 (44.7) | 7524 (57.2) | 6744 (35.9) |  |
| Never married | 4676 (14.6) | 2686 (20.4) | 1990 (10.6) |  |
| Living with partner | 1292 ( 4.0) | 793 (6.0) | 499 (2.7) |  |
| Other status | 6103 (19.1) | 2144 (16.3) | 3959 (21.1) |  |
| NA | 5594 (17.5) | 0 (0) | 5594 (29.8) |  |
| **PIR, Mean ± SD** | 2.6 ± 1.6 | 2.9 ± 1.6 | 2.1 ± 1.5 | < 0.001 |
| **PA (IQR)** | 180.0 (0.0, 840.0) | 300.0 (31.5, 1200.0) | 120.0 (0.0, 540.0) | < 0.001 |
| **Smoke, n ( %)** | | | | < 0.001 |
| Never | 14,480 (55.0) | 7714 (58.7) | 6766 (51.3) |  |
| Former | 6454 (24.5) | 2723 (20.7) | 3731 (28.3) |  |
| Now | 5413 (20.5) | 2710 (20.6) | 2703 (20.5) |  |
| **CHD, n ( %)** | | | | < 0.001 |
| No | 25,123 (78.7) | 12,931 (98.4) | 12,192 (64.9) |  |
| Yes | 1130 ( 3.5) | 216 (1.6) | 914 (4.9) |  |
| NA | 5680 (17.8) | 0 (0) | 5680 (30.2) |  |
| **Angina, n ( %)** | | | | < 0.001 |
| No | 25,552 (80.0) | 13,022 (99.0) | 12,530 (66.7) |  |
| Yes | 714 ( 2.2) | 125 (1.0) | 589 (3.1) |  |
| NA | 5667 (17.7) | 0 (0) | 5667 (30.2) |  |
| **Hypertension, n ( %)** | | | | < 0.001 |
| No | 15,808 (49.5) | 9403 (71.5) | 6405 (34.1) |  |
| Yes | 10,536 (33.0) | 3744 (28.5) | 6792 (36.2) |  |
| NA | 5589 (17.5) | 0 (0) | 5589 (29.8) |  |

CHD, coronary heart disease; IQR, interquartile range; NA, not available; PIR, poverty income ratio; PA, physical activity; SD, standard deviation.
